# Vitamin B Complex and Experimental Autoimmune Encephalomyelitis –Attenuation of the Clinical Signs and Gut Microbiota Dysbiosis

**DOI:** 10.3390/nu14061273

**Published:** 2022-03-17

**Authors:** Marija Mandić, Katarina Mitić, Predrag Nedeljković, Mina Perić, Bojan Božić, Tanja Lunić, Ana Bačić, Mirjana Rajilić-Stojanović, Sanja Peković, Biljana Božić Nedeljković

**Affiliations:** 1 Institute of Physiology and Biochemistry “Ivan Đaja”, Faculty of Biology, University of Belgrade, 11000 Belgrade, Serbia; marija.mandic@bio.bg.ac.rs (M.M.); katarina.mitic@bio.bg.ac.rs (K.M.); minap@bio.bg.ac.rs (M.P.); bbozic@bio.bg.ac.rs (B.B.); tanja.lunic@bio.bg.ac.rs (T.L.); 2 Department for Plastic and Reconstructive Surgery, Institute for Orthopedic Surgery “Banjica”, 11000 Belgrade, Serbia; nedeljkovicpredrag@gmail.com; 3 Laboratory for Human Molecular Genetics, Institute of Molecular Genetics and Genetic Engineering, 11000 Belgrade, Serbia; 4 Faculty of Technology and Metallurgy, University of Belgrade, 11000 Belgrade, Serbia; abacic@tmf.bg.ac.rs (A.B.); mrajilic@tmf.bg.ac.rs (M.R.-S.); 5 Department of Neurobiology, Institute for Biological Research “Siniša Stanković”, National Institute of Republic of Serbia, University of Belgrade, 11060 Belgrade, Serbia; sanjapekovic@gmail.com

**Keywords:** vitamin B complex, experimental autoimmune encephalomyelitis, nerve/muscle nuclear density, popliteal lymph nodes, gut microbiota, neuroprotection

## Abstract

The present study aimed to investigate the neuroprotective effects of the vitamin B complex (B1, B2, B3, B5, B6, and B12—VBC), by studying the changes in the femoral nerve, quadriceps muscle, popliteal lymph nodes and gut microbiota in the rat model of multiple sclerosis, experimental autoimmune encephalomyelitis (EAE). VBC treatment attenuated clinical signs of EAE during the disease, and reduced the duration of EAE thereby contributing to a faster recovery. In VBC-treated EAE rats, a significant decrease in nerve and muscle nuclear density was revealed during the onset period of the disease, while a marked increase was detected at the end of the disease, compared with untreated EAE rats. In the lymph nodes of VBC-treated EAE rats, a fewer number of lymphoid follicles in the cortical area and smaller epithelioid granulomas were detected. The changes in microbiota composition were examined using 16S rRNA gene sequencing and bioinformatics analysis, which revealed the potential of VBC treatment in establishing and/or maintaining gut microbiota homeostasis. Finally, the present study demonstrated that VBC treatment ameliorated the cellular changes in the affected peripheral nerve, muscles innervated by this nerve, and the gut microbiota dysbiosis which occurred during the EAE.

## 1. Introduction

Experimental autoimmune encephalomyelitis (EAE) represents one of the most frequently used experimental models of multiple sclerosis (MS) [1,2] that mimics many aspects of MS symptoms and pathology [3]. In humans, MS is a neuroinflammatory, demyelinating and T lymphocytes mediated disease of the central nervous system (CNS) [4]. The principal pathological hallmarks of MS include neuroinflammation, demyelination (myelin sheath loss and damage), axonal deterioration, and gliosis (glial cells reaction to CNS impairment) [1,5]. The experimental model of MS can be established either via active immunization with an adjuvant consisting of myelin-derived proteins or peptides, such as myelin basic protein, proteolipid protein, and myelin oligodendrocyte glycoprotein [6,7] or the passive transfer of activated myelin-specific CD4+ T lymphocytes [8]. MS is mediated by Th1, as well as Th17 CD4+ T lymphocytes [9], and is manifested as neuronal deficiency followed by relapsing/remitting phase. As observed in MS, EAE is characterized by demyelination and infiltration of different immune cells in CNS [1].

The unfavorable changes in gut microbiota composition, known as “dysbiosis’’, directly modulate the immune response and therefore presumably represent the fundamental factor for the development of various inflammatory diseases [10]. The gut microbiota is essential for proper maturation of immune cells and maintenance of immune homeostasis, regulating both pro- and anti-inflammatory responses [11]. Additionally, the gut microbiota influences distant tissues, including the CNS [12]. It has been demonstrated that gut microbiota communicates with the CNS along the bidirectional gut-brain axis, having an impact on the permeability of the blood-brain barrier (BBB), hence affecting neural regulation pathways and CNS homeostasis [13]. Numerous studies have shown that gut microbiota dysbiosis plays a significant role in different diseases including MS [14], as well as in EAE [15]. Namely, it was shown that microbiota metabolites are key players in the regulation of immune response, via several pathways mediated by different microbial metabolites including short chain fatty acids, tryptophan metabolites, polysaccharide A, etc., [16]. For instance, it was demonstrated that short chain fatty acids, including propionate and butyrate, produced by certain bacteria, can induce the differentiation of naive CD4+ T lymphocytes into T regulatory lymphocytes, and therefore directly influence the T-helper/T-regulatory ratio which is a crucial element in immune-mediated diseases, such as MS [16,17]. More specific for MS pathology, a recent study identified p-cresol sulphate, indoxyl sulphate and N-phenylacetylglutamine (bacterial metabolites of tryptophan and phenylalanine) as neurotoxic mediators of gut–brain communication in MS [18]. Interestingly, it was shown that germ-free or gnotobiotic mice, did not develop EAE, indicating that the microbiota is required for disease induction [19].

Vitamins of the B group are water-soluble vitamins with many beneficial effects on the nervous system, which deficiency has been linked to different neurodegenerative diseases [20]. As demyelination and axonal deterioration are common for both vitamin B deficiency and many neurological diseases, including MS, it is believed that vitamin B treatment could aid in axonal recovery and improvement of neuronal function [21,22,23,24]. In our previously published paper [25], we have shown that VBC (a complex of vitamins B1, B2, B3, B5, B6, and B12) treatment enhanced the motor nerve regeneration and the recovery of muscle function in a rat model of peripheral nerve injury. Briefly, VBC therapy improved axonal regeneration, as well as functional recovery, by reducing both Schwann cell decline and deterioration of myelin sheath [26]. Moreover, we have demonstrated that treatment with VBC complex accelerated this recovery via attenuation of neuroinflammation [27]. In addition, it was shown that the vitamins B produced by gut microbiota are important for host health but also for bacterial colonization as well as in regulating the immune response [28]. Vitamins B are used by gut microbiota as cofactors of numerous metabolic pathways, playing an important role in regulating the gut microbiota homeostasis [28]. On the other hand, gut dysbiosis could lead to a decrease in vitamin B levels, causing disruption of the immune homeostasis and further gut dysbiosis development allowing for colonization by pathogenic strains. Therefore, the modification of gut microbiota using VBC may provide an adjuvant therapeutic approach for controlling MS progression by promoting anti-inflammatory response and consequential reduction of CNS inflammation.

As a part of our investigation of the VBC effect on neuroinflammation/neuroreparation [27], the objective of this study was to investigate whether VBC could ameliorate clinical signs that occur during EAE. The effects of VBC treatment on affected peripheral nerve and innervated muscle, processes in draining lymph node, and gut microbiota in the EAE rat model were evaluated at different time points of the disease course (onset, peak, and end).

## 2. Materials and Methods

### 2.1. Animals

Male Dark Agouti (DA) rats aged between 2 and 2.5 months (weighing between 220–250 g) were obtained from the breeding colony at Military Medical Academy, Belgrade, Serbia. The animals were maintained at the animal facility of the Faculty of Biology (University of Belgrade, Belgrade, Serbia), in standard macrolone cages (3 rats/cage) under conventional conditions: constant temperature and humidity, 12 h light/dark cycle, with free access to food pellets and tap water. Paralyzed animals were fed and given water manually. Ethics Review Committee for Animal Experimentation of Military Medical Academy and Ministry of Agriculture and Environmental Protection Republic of Serbia, Veterinary Directorate No. 323-07-6180/2019-05 and No. 323-07-06858/2021-05, approved all the experiments conducted on animals. The experiments were performed in compliance with the EEC Directive (2010/63/EU) on the protection of animals used for experimental and other scientific purposes.

### 2.2. Chemicals

Vitamin B complex (Beviplex^®^ ampoule containing B_1_ (40 mg), B_2_ (4 mg), B_3_ (100 mg), B_5_ (10 mg), B_6_ (8 mg), and B_12_ (4 μg)) was obtained from Galenika a.d. Belgrade, Serbia. Complete Freund’s adjuvant, containing 1 mg/mL *Mycobacterium tuberculosis* (CFA) was obtained from Sigma-Aldrich (St. Louis, MO, USA).

### 2.3. Induction and Treatment of Experimental Autoimmune Encephalomyelitis (EAE)

**Figure 1:**
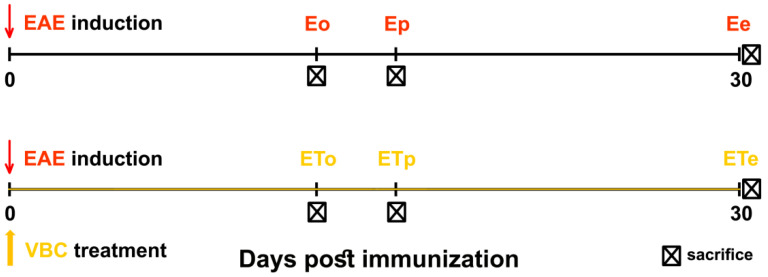
**Illustration of the experimental design and time points of animal sacrifice**. Untreated EAE rats (**E**), VBC-treated EAE rats (**ET**), onset (**o**), peak (**p**), and end (**e**) time points.

Animals (n = 47) were randomly categorized into four different experimental groups: control intact group (**C**, n = 6); group subcutaneously injected only with Complete Freund’s adjuvant (**CFA**, n = 6); untreated EAE group (**E**, n = 17); VBC-treated EAE group (**ET**, n = 18). The animals from **E** and **ET** experimental groups were sub-divided and studied at three-time points: onset (**o**), peak (**p**), and the end (**e**) of EAE (**Eo**, **Ep**, **Ee** and **ETo**, **ETp**, **ETe**, respectively). EAE was induced by subcutaneous injection (into the left hind footpad) of 150 μL of an emulsion containing rat spinal cord homogenate (50% *w/v* in saline) in Complete Freund’s adjuvant. Immunization was performed under ether anesthesia. Subcutaneous injection of Complete Freund’s adjuvant of equal volume was administrated in age-matched rats in the **CFA** group. In the **ET** experimental group, animals were treated with VBC which was dissolved in saline and administered intraperitoneally once per-day (*i.p.* 1.85 mL/kg body weight/day) starting from the onset of immunization (day 0) until the end of the experiment (day 30), while EAE-untreated rats (**E** group) received the equal volume of saline. The experimental design is given in Figure 1.

### 2.4. Clinical Assessment of EAE

The rats were daily examined, weighed and scored for neurological signs of EAE during 30 days after immunization, by two independent observers according to standard 0–5 EAE grading scale: 0, no clinical signs; 0.5, partial loss/reduced tail tone assessed by the inability to curl the distal end of the tail; 1, flaccid tail; 1.5, slightly/moderately clumsy gait, impaired righting ability, or combination; 2, hindlimb weakness; 2.5, partial hindlimb paralysis; 3, complete hindlimb paralysis; 3.5, complete hindlimb paralysis and forelimb weakness; 4, quadriplegia; and 5, moribund state or death. The monitoring and scoring procedure was used for all animals. The mean clinical scores for animals in each experimental sub-group were calculated at the end of the evaluation period (day 30).

### 2.5. The Determination of Nerve/Muscle Nuclear Density and Histological Examination of Popliteal Lymph Nodes

After the animals were sacrificed by decapitation, they were positioned for dissection of femoral nerves and quadriceps muscles of both back legs. Moreover, popliteal lymph nodes, located in the lymph drainage route, were collected. The quadriceps muscles, femoral nerves, and popliteal lymph nodes were washed in saline solution and prepared for histological H&E technique (Laboratory Oculus, Belgrade, Serbia). A series of muscles, nerves, and lymph nodes sections were prepared for histological assessment. Afterwards, nerve and muscle samples were inspected under a 40× magnification, while lymph nodes samples were observed under 5×, 10×, and 40× magnification of the brightfield microscope (Leica, LAS V4.11 software was used for imaging). ImageJ software was utilized for nuclei counting and determination of nuclear density (nuclei per mm^2^).

### 2.6. Tissue Collection and DNA Extraction for 16S rRNA GENE Sequencing

For each time point of the disease course (**o**, **p**, and, **e**), fecal samples of each animal were collected in all (sub-)groups (**C**, **CFA**, **Eo**, **Ep**, **Ee**, **ETo**, **ETp**, and **ETe**). The genomic DNA was isolated using the Sigma-Aldrich GenEluteTM Stool DNA Isolation Kit according to the manufacturer instructions. Briefly, lysates were prepared by adding lysis buffer to the app. 80 mg of each sample. Following the centrifugation steps, supernatant (700 µL) was collected and equal volume of 70% ethanol was added and DNA was eluted. Purified DNA was stored at −20 °C for a longer period time. Quality control of isolated DNA was evaluated by 1% agarose gel electrophoresis. Sample for each (sub-)group was prepared for 16S rRNA gene sequencing (pooled DNA of each animal in equal portion). 

### 2.7. 16S rRNA Protocol

Gut microbiota was assessed by 16S rRNA gene amplicon sequencing, performed using the Illumina NovaSeq 6000 platform. PCR amplification of (sub-)group genomic DNAs was performed using barcoded primers targeting the hypervariable V4-V5 region of the 16S rRNA gene: 515F (5′-GTG CCA GCM GCC GCG GTA A-3′) and 907R (5′-CCG TCA ATT CCT TTG AGT TT-3′). A total of 672,480 sequencing reads were generated from eight samples, with an average of 84,060 ± 14,184 reads per sample. The raw data were processed using the programming language R version 4.1.2 [29] and using the Bioconductor software version 3.14 [30]. Raw sequencing reads were filtered and trimmed to a length of 200 base pairs, following the quality control assessment. The DADA2 pipeline was used for the denoising step and to generate the amplicon sequence variants (ASVs) [31]. After running the DADA2 inference algorithm, the sequences were checked for the presence of chimeras and chimeric sequences were removed. A total of 476,009 reads parsed the upstream analysis with the range of 42,311–77,952 reads per sample (average number of reads 61,554 ± 10,924). Generated ASVs were aligned against the SILVA 138 reference database for the phylogenetic identification [32]. Further, microbiota analysis was performed using the Microeco package (v.0.6.5) and the relative abundances of ASVs were used in the construction of the data tables from phylum to genus level [33]. The singletons and the taxa with the relative abundance lower than 0.1% were removed from the analysis and the differences in microbial composition among the experimental groups were examined.

Microbial community diversity within and between samples was assessed by measuring alpha and beta diversity indices. Shannon diversity index was calculated as a measure of alpha diversity, whereas beta diversity was evaluated by performing weighted UniFrac distances. Log transformed weighted UniFrac distances between samples were used to perform a principal coordinate analysis (PCoA) plot. Correlation between the relative abundance of 50 most abundant microbial genera and the clinical score was assessed by calculating Pearson correlation coefficient. Differentially abundant phylogenetic groups were detected by performing the differentially abundance test based metastat method implemented in the Microeco package.

### 2.8. Statistical Analysis

Descriptive statistics were presented as mean values with standard error (SE). Student t-test and one-way ANOVA, followed by Fisher’s PLSD test were used to calculate differences between mean values. A *p* < 0.05 was considered as significant. Statistical analysis was performed using the SPSS software for Windows, version 20.0 (SPSS, Inc., Chicago, IL, USA).

## 3. Results

### 3.1. VBC Treatment Attenuates Severity and Duration of EAE

**Figure 2:**
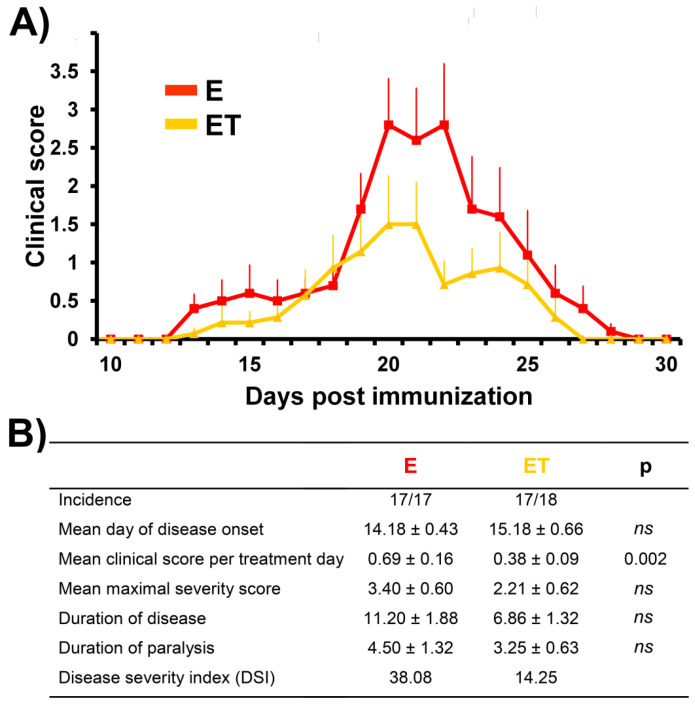
**Clinical scores of untreated EAE rats (E) vs. VBC-treated EAE rats (ET) during the disease course.** (**A**) The mean clinical scores of rats were monitored daily and the animals were sacrificed as described in Materials and Methods. Animals in **E** group were immunized and 150 μL saline was applied daily as *i.p.* injection from the onset of immunization (0 dpi) until the end of experiment (30 dpi). Animals in **ET** group were immunized and treated with cocktail of B vitamins (B1, B2, B3, B5, B6, B12) at a dosage 1.85 mL/kg body weight/every day, *i.p.*, from the onset of immunization (0 dpi) to 30 following days (end of experiment). (**B**) The effect of VBC treatment on parameters of the disease. Data are expressed as mean ± standard error (SE), *ns*—nonsignificant.

Conventional immunization protocol with rat spinal cord emulsified in CFA usually induces EAE in DA rats, highly susceptible strain to development of EAE. In the present study, all immunized rats in the **E** group (17/17) developed the acute monophasic disease, while in the **ET** group 17/18 developed clinical signs of the EAE. The animals from **C** and **CFA** groups did not develop EAE (0/6 per group). In the **E** group the first signs of EAE appeared at 14 ± 0.43 days post-immunization (dpi), while rats from the **ET** group have shown the first clinical signs of EAE slightly, and not significantly later at 15 ± 0.66 dpi (Figure 2).

The symptoms peaked at 18 ± 0.95 dpi in the **E** group, while in the **ET** group peak of disease was detected at 19 ± 0.98 dpi. Clinical signs of EAE decreased afterwards during the period of recovery, and all rats in the **E** group completely recovered until 26 ± 0.76 dpi while in the **ET** group animals completely recovered until 23 ± 0.89 dpi (end of disease). Parameters of the disease were evaluated throughout the disease (Figure 2B), including incidence (a number of rats that developed any clinical sign); mean day of disease onset (the mean day on which afflicted animals developed the first clinical sign); mean clinical score per treatment day (mean disease symptoms for all rats within a given group per day); mean maximal severity score (the mean of maximal clinical score that each rat developed throughout the disease); duration of disease (the mean number of days the rat had any symptoms); duration of paralysis (the mean number of days the rat had paralysis throughout the experiment); disease severity index (DSI = (maximal severity of the disease) × (duration of the disease) × (incidence)). Rats treated with VBC (**ET**) developed a milder form of the disease (DSI = 14.25) in comparison to rats of the **E** group (DSI = 38.08). Additionally, VBC treatment has led to a shorter duration of disease (6.86 ± 1.32 vs. 11.20 ± 1.88 days, respectively, **ET** vs. **E**), although this difference was at the border of significance (*p* = 0.079, Figure 2B). Similarly, the mean maximal severity score in the **ET** group was lower when compared to the **E** group (2.21 ± 0.62 vs. 3.40 ± 0.60, respectively, **ET** vs. **E**), while the mean clinical score per treatment day was significantly (*p* < 0.002) smaller in the treatment group (0.38 ± 0.09 vs. 0.69 ± 0.16, respectively, **ET** vs. **E**) (Figure 2B). It should be emphasized that although it was not statistically significant VBC treatment decreased body weight loss during the peak of disease which is in line with lower clinical scores observed (Figure S1).

### 3.2. Histological Examination of Popliteal Lymph Nodes as Drain Lymph Nodes after Induction of EAE

**Figure 3:**
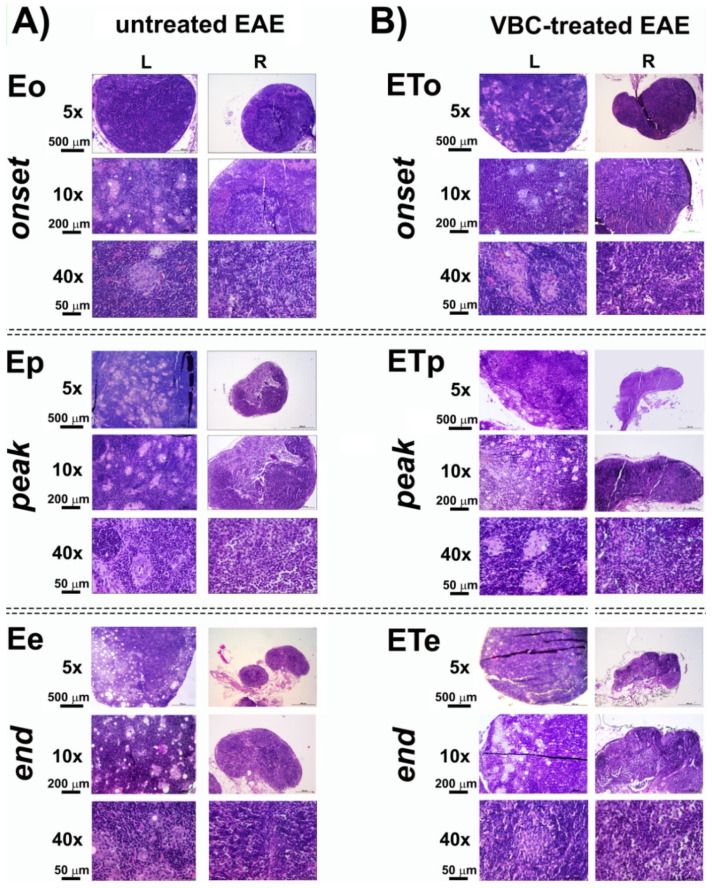
**Histological changes in popliteal lymph nodes structure of untreated EAE rats** (**A**) **and VBC-treated EAE rats** (**B**). Micrographs of transverse sections of H&E stained lymph nodes (left—**L** and right—**R**) in three different time points: onset (**o**), peak (**p**), and end (**e**) of the disease. Magnification 5× (scale bar 500 μm), 10× (scale bar 200 μm), and 40× (scale bar 50 μm).

Histological examinations of the popliteal lymph node (LN), as a drain lymph node, section of both control groups (**C** and **CFA**) are shown in Figure S2. The cortex contained several primary lymphoid follicles, mainly poorly developed without visible germinal centers, which underwent intensive staining (Figure S2). The histology of LN isolated from untreated EAE rats (**E**) is shown in Figure 3. The LN in the rats from this group, isolated at the time point marked as the disease onset (**Eo**), showed a decrease in the cortex thickness with medullary hyperplasia (Figure 3A), secondary lymphoid follicles with prominent germinal center and small epithelioid granulomas (Figure 3A).

The histological examination of popliteal LN sections from untreated EAE animals at the peak of disease (**Ep**), showed the masses of macrophages distributed throughout the whole section, which extend to the medulla. Large epithelioid granulomas were observed on high magnification 40×, containing epithelioid cells forming granuloma, surrounded by a collar of lymphocytes, accompanied by cortical atrophy (Figure 3A). Similar observations were detected on sections from animals at the end of the disease (**Ee**), but with a fewer number of secondary lymphoid follicles with a germinal center and small epithelioid granulomas (Figure 3A). Histological examinations of sections obtained from EAE animals treated with VBC showed a fewer number of lymphoid follicles in the cortical area during the **ETo**, while smaller epithelioid granulomas during **ETp** and **ETe** time points were detected (Figure 3B). Additionally, a significant difference was detected between the size/extent of left LNs in comparison with right LNs after EAE induction (Figure S3).

### 3.3. Nerve Nuclear Density

**Figure 4:**
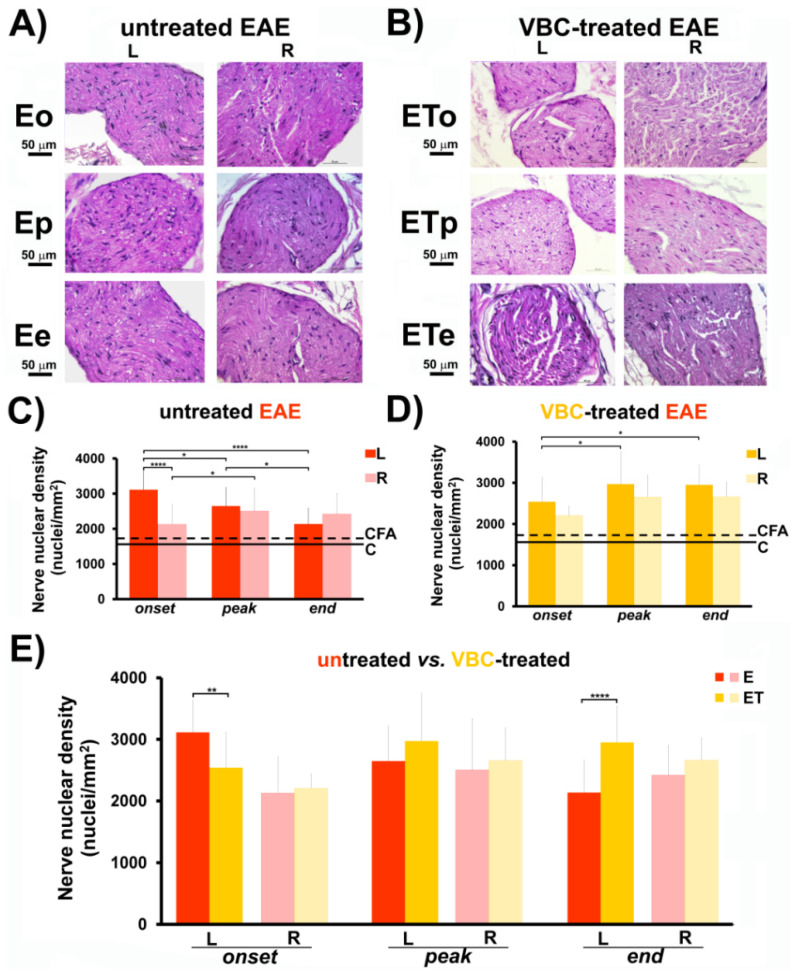
**The effects of VBC treatment on nerve nuclear density during EAE.** (**A**) Histological changes of nerve nuclear density in untreated EAE rats (**E**). Scale bar 50 μm (Magnification 40×). (**B**) Histological changes of nerve nuclear density in VBC-treated EAE rats (**ET**). Scale bar 50 μm (Magnification 40×). (**C**) Changes in nerve nuclear density in untreated EAE rats (**E**). (**D**) Changes in nerve nuclear density in VBC-treated EAE rats (**ET**). (**E**) Changes in nerve nuclear density compared in both groups (**E** and **ET**). The nuclear density is monitored in three different time points: onset (**o**), peak (**p**), and end (**e**) of the disease. Control intact rats (**C**), rats *s.c.* injected only with Complete Freund’s adjuvant (**CFA**). Left (**L**) and right (**R**) nerve. Results are presented as the mean ± standard error (SE). **** *p* < 0.0001; ** *p* < 0.01; * *p* < 0.05.

The nuclear densities analysis of isolated femoral nerves at three different time points showed that in the untreated group of EAE rats this feature has changed significantly more evidently than in the treated group (Figure 4C–E). Animals with EAE from both treated and untreated groups (**E** and **ET**) showed a significant increase in nerve nuclear density in comparison to the control groups (**C** and **CFA**, *p* < 0.01). Regarding the EAE untreated group, at the onset time point (**Eo**), the left femoral nerve displayed a notable rise in nuclei number/mm^2^ (3112 ± 523) when compared to the right femoral nerve (2127 ± 576, *p* < 0.001, Figure 4C). During the disease peak and at the disease end, there was no significant difference between left and right femoral nerve in either **E** or **ET** groups (Figure 4C,D). In addition, for different time points (**Eo** vs. **Ep/Ee**), prominent alterations in nuclei number/mm^2^ were noted for both groups (**E** and **ET**) (Figure 4C,D). Moreover, in Figure 4E, it can be observed that nuclei/mm^2^ of left femoral nerve of the **ET** group (at time point **ETo**) showed a decrease in number (2540 ± 594) compared to the left femoral nerve of the **E** group at onset (3112 ± 523, *p* < 0.01). In contrast, at the end of the disease (**ETe**), an increase in nuclei number of the left femoral nerve was observed in the **ET** (2950 ± 480) compared to the **E** group (2136 ± 451, *p* < 0.001, Figure 4E). Micrographs of H&E stained femoral nerve transverse sections, showing marked changes of nuclear density, are separately presented (Figure 4A,B). Histological examinations of nerve nuclear density of both control groups (**C** and **CFA**) are shown in Figure S4.

### 3.4. Muscle Nuclear Density

**Figure 5:**
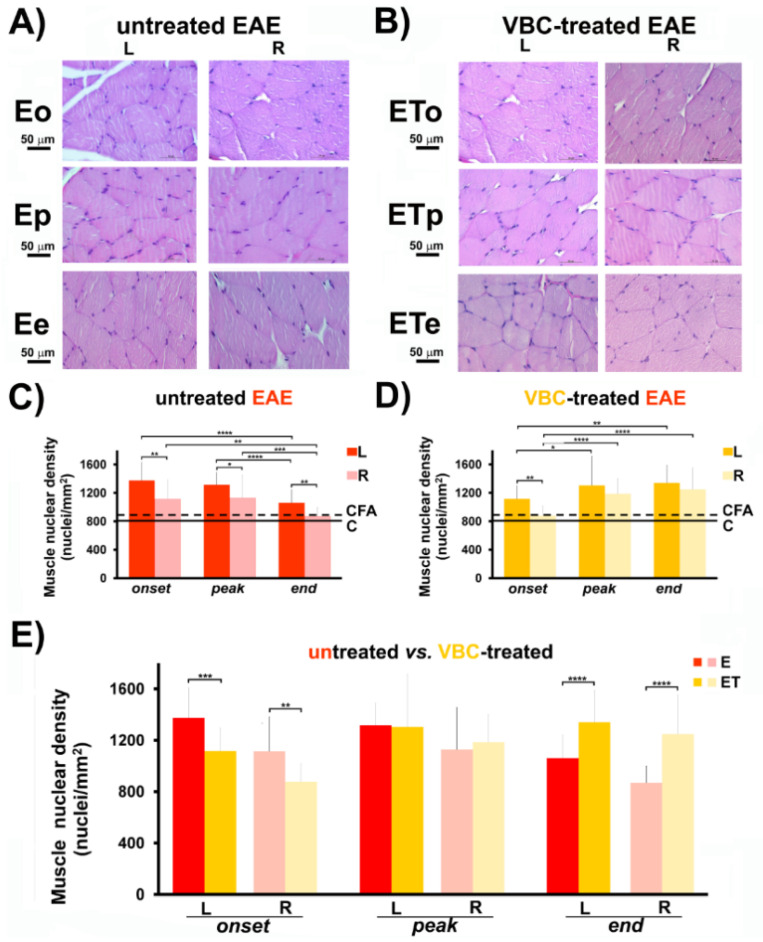
**The effects of VBC treatment on muscle nuclear density during EAE.** (**A**) Histological changes of muscle nuclear density in untreated EAE rats (**E**). Scale bar 50 μm (Magnification 40×). (**B**) Histological changes of muscle nuclear density in VBC-treated EAE rats (**ET**). Scale bar 50 μm (Magnification 40×). (**C**) Changes in muscle nuclear density in untreated EAE rats (**E**). (**D**) Changes in muscle nuclear density in VBC-treated EAE rats (**ET**). (**E**) Changes in muscle nuclear density compared in both groups (**E** and **ET**). The nuclear density is monitored in three different time points: onset (**o**), peak (**p**), and end (**e**) of the disease. Control intact rats (**C**), rats *s.c.* injected only with Complete Freund’s adjuvant (**CFA**). Left (**L**) and right (**R**) muscle. Results are presented as the mean ± standard error (SE). **** *p* < 0.0001; *** *p* < 0.001; ** *p* < 0.01; * *p* < 0.05.

The nuclear densities of isolated quadriceps muscles at different time points for both groups (**E** and **ET**) showed a significant increase (*p* < 0.0001) in muscle nuclear density in comparison with control groups (**C** and **CFA**). For all time points in the **E** group and onset time point in the **ET** group, a significant increase in nuclear density in the left muscle compared to the right muscle was detected (Figure 5C,D). There was no significant increase in muscle nuclear density between left and right muscle for **ETp** and **ETe** groups (Figure 5D). Interestingly, muscle nuclear density alterations (Figure 5E) followed the same trend of change as nerve nuclear density (Figure 5E). Namely, at the disease onset, a decrease of nuclei number/mm^2^ (1116 ± 180) was observed in the left muscle of **ET** group, compared to the left muscle of the **E** group (1375 ± 255), while, at the disease end, a notable increase in nuclei number/mm^2^ (1341 ± 250) was detected in the left muscle of the **ET** group, compared to the left muscle of **E** group (1061 ± 181), (Figure 5E). In parallel, the same data trend was detected for the nuclei number/mm^2^ for the right muscles of both aforementioned animal groups (Figure 5E). Micrographs of H&E stained quadriceps muscle transverse sections, showing prominent changes of nuclear density, are separately presented (Figure 5A,B). Histological examinations of muscle nuclear density of both control groups (**C** and **CFA**) are shown in Figure S4.

### 3.5. Gut Microbiota Analysis

To characterize the influence of VBC therapy on gut microbiota composition during EAE, genomic DNA was extracted from fecal samples of rats from each experimental (sub-)group. Using this material, the bacterial microbiota composition of each (sub-)group was assessed by 16S rRNA gene sequencing. There was no significant difference in alpha diversity between the samples. However, frequently analyzed marker of microbiota–Firmicutes to Bacteroidetes ratio (F:B) was found to be increased in untreated EAE animals during the disease (onset, peak, and end; 0.42, 0.33, and 0.33, respectively), compared to intact control animals (F:B ratio of 0.13). In VBC-treated EAE animals, F:B ratio also increased compared to the intact control animals (onset, peak, and end; 0.38, 0.30, and 0.22, respectively), although with lower F:B ratio values observed for the untreated EAE animals. These data indicated that VBC treatment may have a role in regulating the microbiota dysbiosis induced by EAE, due to the prominent decrease in F:B ratio at the end of the disease.

**Figure 6:**
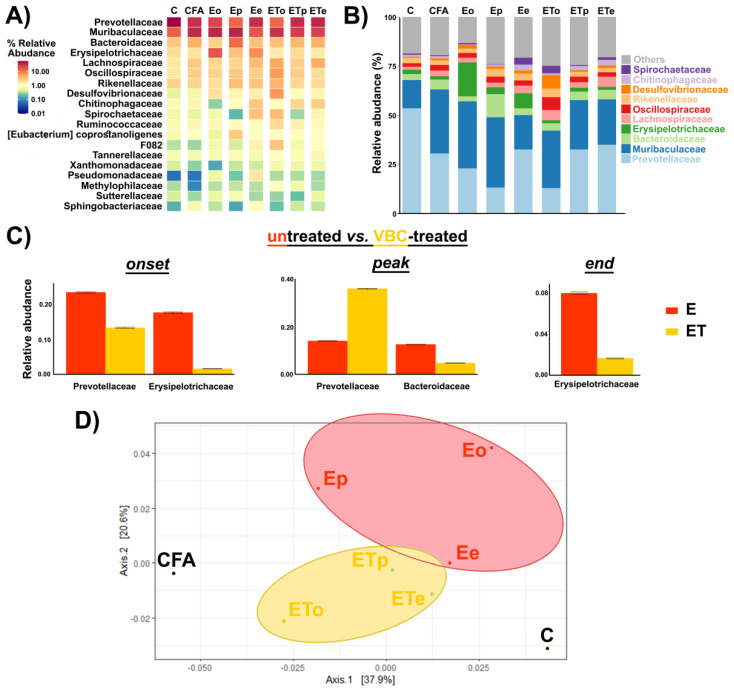
**Gut microbiota analysis—**(**A**) The abundance-based heatmap of 20 most abundant bacterial families level. (**B**) Relative abundances (%) of top 10 identified bacterial families. (**C**) Differentially abundant families between untreated and VBC-treated animals based on metastat method. (**D**) Principal component analysis (PCA) and clustering of the gut bacterial communities based on log transformed weighted Unifrac distance. Untreated EAE rats (**E**), VBC-treated EAE rats (**ET**) in three different time points: onset (**o**), peak (**p**), and end (**e**) of the disease, control intact rats (**C**), and rats *s.c.* injected only with Complete Freund’s adjuvant (**CFA**). Percentage values in parentheses represent a percentage of variance explained by each component.

The abundance-based heatmap analysis at the family level (Figure 6A), as well as the bar chart showing the taxonomic abundance at the family level, revealed clear differences between bacterial composition in VBC-treated (**ET**) and untreated (**E**) EAE animal groups. At the family level, the top 10 bacteria were identified and included Prevotellaceae, Muribaculaceae, Bacteroidaceae, Erysipelotrichaceae, Lachnospiraceae, Oscillospiraceae, Rikenellaceae, Desulfovibrionaceae, Chitinophagaceae, and Spirochaetaceae (Figure 6B). According to the data presented in Figure 6B, the most prominent changes were the decline in Prevotellaceae and the increase in the Muribaculaceae family in all groups when compared to the intact control animals (**C**). At the peak of the disease in the untreated animal group (**E**), a significant rise of the Bacteriodaceae family was observed while in the treatment group (**ET**) it remained consistent during all time points of the disease (Figure 6B). Additionally, a significant elevation of the Erysipelotrichaceae family was noted in the **E** group at the onset and the end of the disease (Figure 6A,B).

According to the metastat analysis (Figure 6C), a significant decrease of the relative abundance of Prevotellaceae and Erysipelotrichaceae was detected during the onset in the VBC-treated (**ET**), compared to untreated (**E**) animal group, while during the peak of the disease, in the VBC-treated animal group, a significant increase in Prevotellaceae remained accompanied with a significant decrease in Bacteroidaceae family abundance. Finally, at the end of the disease, significant differences between **E** and **ET** groups were detected only for the Erysipelotrichaceae family.

Principal component analysis showed clear segregation of gut microbiota from VBC-treated and untreated EAE animals, which was further separated from the microbiota of the controls (Figure 6D). Interestingly, the microbiota of EAE animals at the end of the treatment, when all animals recovered, was the most similar to the microbiota of the control group, suggestive of the significant role of gut microbiota in the recovery process.

The weighted Unifrac distance calculated between the control and all other samples (Supplementary, Table S1) indicated that the microbiota of VBC-treated EAE animals at the end (**ETe**) of the trial was the most similar to the microbiota of the control group (**C**). In addition, during the disease, the calculated distance was decreasing at two-fold higher rate for the VBC-treated group when compared to the untreated group (dissimilarity decrease of 8.3 vs. 4.5 between time points for **ET** and **E** group, respectively).

**Figure 7:**
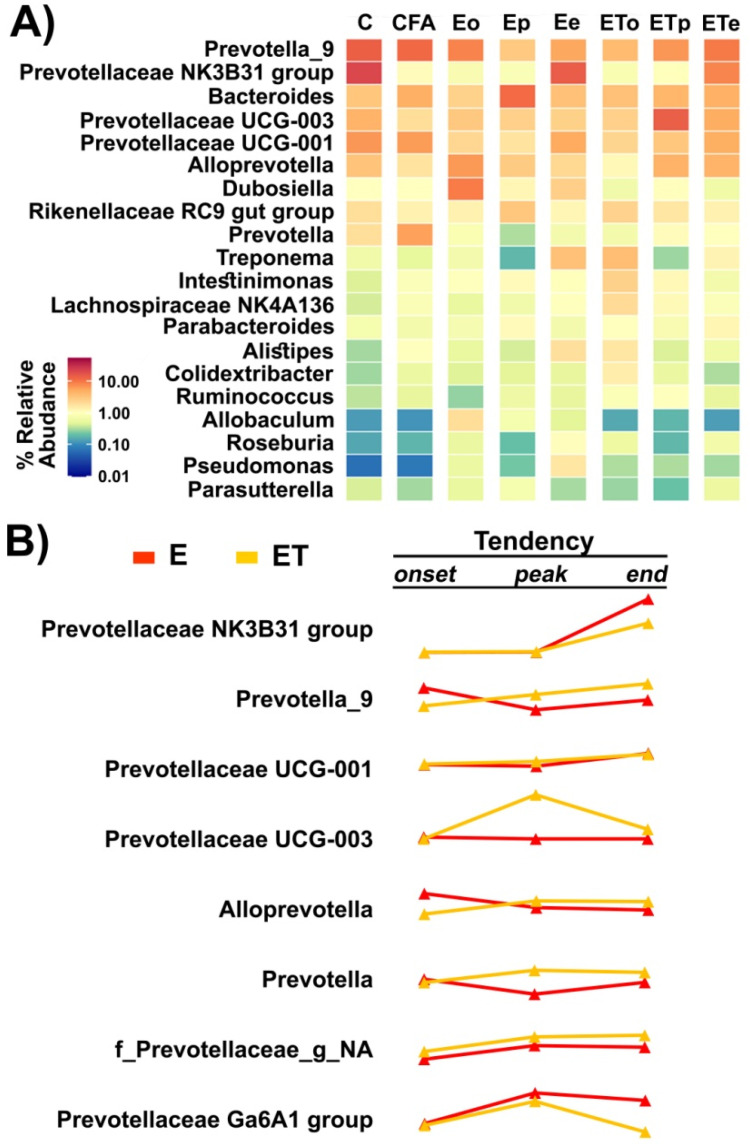
**Gut microbiota analysis at the Genus level—**(**A**) The abundance-based heatmap of the top 20 bacterial genera (**B**) The trend of change of relative abundance of genera within the Prevoteallaceae family for VBC-treated and untreated EAE animals during the course of the disease.

Several *Prevotella* genera were identified as highly abundant in different samples, while differential abundance was detected for a number of pairs of comparisons (Figure 7A). Differentially abundant Prevoteallaceae included *Prevotellaceae* NK3B31 group, *Prevotella*_9, *Prevotellaceae* UCG-001, and *Prevotellaceae* UCG-003 and there was a notable change in the abundance of these groups throughout the disease course. A significant negative correlation between the abundance of *Prevotellaceae* UCG-001 and clinical score (Pearson correlation coefficient −0.719, *p* value 0.044) showed that alleviation of EAE symptoms was either followed or induced by the increase in the abundance of this bacterial group. Finally, it was noted that the abundance ratio of *Prevotella* at the end point when animals did not show symptoms of EAE, in both **E** and **ET** groups, were similar to those observed for the control group (**C**). The trend of change of relative abundance of genera within the Prevoteallaceae family (Figure 7B) was presented according to the relative abundance values shown in Table S2.

## 4. Discussion

B vitamins are essential for diverse metabolic pathways and cellular reactions [22,34]. They are required for the metabolism of carbohydrates, proteins, and lipids, and also play key roles in the regulation of many enzymatic reactions, in the form of coenzymes. Most importantly, vitamins of B group are fundamental in providing the energy for the proper development of the nervous system. Their deficiency has been associated with cognitive dysfunction and neurological diseases such as Parkinson’s, Alzheimer’s disease, and MS [22,34]. Importantly, B vitamins are produced by gut microbiota and their altered metabolism has been linked to gut dysbiosis which occurs in neurodegenerative diseases [35].

The results of the present study revealed that VBC treatment affected the course of the disease at specific time points, following the EAE induction. VBC caused a reduction in the number of lymphoid follicles in the cortical area, while smaller epithelioid granulomas were observed in the treated EAE rats. At the early phase of the disease, VBC decreased the nerve and muscle nuclear densities, although the significant elevation of nuclear densities of both investigated tissues was noticed at the end of the disease. In addition, it should be emphasized that VBC treatment attenuated clinical signs during the peak and end phase of EAE indicating that rats treated with VBC developed a milder form of the disease accompanied by faster recovery during the period of post-immunization. Finally, VBC treatment in EAE had an impact on the gut microbiota composition.

Interestingly, we detected a significant difference between the size/extent of the left popliteal LNs in comparison with the right popliteal LNs after EAE induction in all animal groups, together with increased nuclear density in femoral nerve and quadriceps muscle isolated from the left legs. Considering that the left leg was challenged for EAE induction, large drain popliteal LNs from this leg could be a consequence of a progressive process of antigen presentation that occurred in these regional draining nodes. Importantly, the applied immunization triggers changes in blood-nerve barrier (BNB) permeability. The maintenance of BNB permeability is regulated by key tight junction proteins, including the family of claudins, occludin, and tricellulin [36]. It has been demonstrated that in rats with EAE, the diminished expression of BNB proteins (i.e., claudin-3 and claudin-5) in the endothelium is mediated by proinflammatory cytokines, TNF-α and IL-17. Disrupted BNB permeability could be a possible explanation for a significant increase of nuclear density/cell recruitment in the femoral nerve and quadriceps muscle, under progressive inflammatory conditions, such as EAE. In other experimental models, the infiltration of mononuclear cells into the sciatic nerve was observed after experimental autoimmune neuritis immunization [37]. In the early phase of VBC treatment of EAE, the changes in nerve function (paresis/paralysis) were followed by a decrease in nerve nuclear density and were observed together with the reduction in muscle nuclear density. On the contrary, our findings displayed that VBC treatment contributed to the increased nerve nuclear density at the end of the disease. These observations could be correlated with our recently published results showing that VBC caused a time-dependent rise of cell number (*i.e.,* macrophages/Schwann cells) and their mutual interactions that are most likely associated with the regeneration of the injured nerve [26,27]. In addition to Schwann cells, one study outlined the significance of the olfactory nerve glia, which is more efficient in removing myelin debris and promoting neuronal regeneration than Schwann cells. Thus, co-transplantation of both cell types has been proposed as a potential therapy for axonal regeneration of the injured nervous system, including CNS and peripheral nervous system (PNS) [38]. The literature data described that VBC, as additional therapy, improved nerve regeneration, by promoting an increase in the number of Schwann cells, which represent one of the most important glial cells of the PNS required for myelin formation and axonal recovery [23,39]. In the present study, the possible explanation for the rise in nuclear density in the affected femoral nerve after EAE induction could be in line with the infiltration process mediated by macrophages of different polarization which facilitate nerve regeneration, by clearing myelin debris and promoting the cell proliferation and migration [40]. One of the most frequently described vitamins of the B group, vitamin B12, was specified as one of the major vitamins responsible for myelin formation. What is more interesting, decreased levels of vitamin B12 have been detected in neuroinflammatory and neurodegenerative conditions, including MS [41,42]. Moreover, in one pilot study [43], it was demonstrated that high doses of B vitamins supplementation (B1, B6, and B12) improved the optical nerve functions in MS patients. Due to their neuroprotective activities, the vitamins of the B complex could represent a potential supplementation therapy for improving the remyelination of affected nerves and their regeneration [24].

In our study, it was detected that VBC treatment contributed to a significant increase in muscle nuclear density at the end of the disease. As previously described [44], denervated muscles show a compensatory myogenic response by forming new muscle fibers from satellite cells in the process that is opposite to muscle atrophy and cell death. The potential mechanism of VBC effects shown in our study could be associated with a compensatory myogenic response and a larger number of satellite cells at the end of EAE. However, a reduced number of counted cells was confirmed in muscles at the day 15 post-immunization, which is in accordance with our previously published results showing the potential of VBC treatment in reducing the muscle nuclear density during neuroinflammation induced by peripheral nerve injury [25].

Various studies have shown the presence of gut microbiota dysbiosis in MS patients [14], as well as in the EAE model [15]. In the present study, we examined the microbiota composition at the onset, the peak, and the end of EAE in both untreated and VBC-treated animals. It was demonstrated that EAE induced gut microbiota shift, especially during the onset and peak of the disease. Interestingly, at the end of the study, when all animals have recovered the microbiota distance from the control was the smallest, indicating a role of microbiota in the recovery process. In addition, the distance between the control and the samples from VBC-treated animals was decreasing more rapidly than for untreated animals. This led us to the conclusion that the beneficial effect of VBC treatment was at least partially mediated by gut microbiota. The most relevant marker of gut microbiota dysbiosis in different pathological conditions [45] is the alteration in F:B ratio. In this study, it was revealed that VBC may have a potential role in regulating the microbiota dysbiosis induced by EAE during all investigated time points. Most importantly, at the end of the disease in VBC-treated animals, F:B ratio was the most similar to the F:B ratio in the intact control animals. Furthermore, it is well established that gut microbiota is a producer of vitamins of B complex [46], and that these vitamins may impact gut microbiota composition and function by, among other means, supporting metabolism of certain bacteria and suppressing colonization of others [47].

The most significant change at the family rank in this study was the decrease in Prevotellaceae abundance in both E and ET groups when compared to the intact control animals. Moreover, analysis of correlation between the disease score and the abundance of top 50 bacterial genera showed a significant negative correlation between *Prevotellaceae* UCG-001 abundance and clinical score, indicating that attenuation of EAE symptoms was either accompanied or induced by the increase in the abundance of this bacteria. *Prevotella* species are anaerobic Gram-negative bacteria belonging to the Bacteroidetes phylum, having a role in polysaccharide breakdown. Numbers of studies pointed out that MS patients had a depleted abundance of *Prevotella* in comparison with healthy subjects, suggesting a potential association between MS and reduced amount of *Prevotella*, as well as the potential role of *Prevotella* in the promotion of anti-inflammatory response [14]. In addition, it was indicated that *Prevotella* stimulates the IL-10 production in the small intestine and that *Prevotella histicola* can suppress EAE [48]. Taken together, it can be suggested that *Prevotella* abundance is in close relationship with disease severity and may play a part in promoting anti-inflammatory response in MS patients. At the peak of the EAE course, *Prevotella* abundance was notably higher in VBC-treated animals when compared to non-treated animals, indicating that the beneficial effect of B vitamins was evident also through reinforcing the gut microbiota homeostasis. Although our data and data of others recognize Prevoteallaceae family relevant for MS pathology, further studies are needed to elucidate if the *Prevotella* genus can be considered as the potential therapeutic target for MS treatment. Our results are in accordance with conclusions of some recent clinical trials [49], which imply that therapeutic interventions which modify the gut microbiota aiming to favor the development of “good” microorganisms with anti-inflammatory action, such as *Prevotella*, might be considered for reduction of the onset of clinical relapses of disease in MS patients.

Furthermore, a notable rise of *Allobaculum* spp. from Erysipelotrichaceae family, and Erysipelotrichaceae family was detected at the onset of EAE. This bacterial family has been shown to participate in the induction of Th17 cell response [50], which could elevate the EAE severity. Both Allobaculum and Erysipelotrichaceae are markers of dysbiosis induced by high fat diets and obesity [51], as well as in inflammation-related gastrointestinal diseases [52]. Moreover, an increase in the abundance of Lachnospiraceae was noted exclusively at the onset of disease in the VBC-treated animals. Recent studies have demonstrated a correlation between Lachnospiraceae and promotion of anti-inflammatory response mediated by IL-10 and TGF-β production by immune cells, accompanied with Treg cell differentiation, which may be linked to detected amelioration of EAE clinical signs in VBC-treated animals [53].

## 5. Conclusions

This study demonstrated that VBC treatment reduced severity and duration of EAE, attenuated changes in draining (popliteal) lymph nodes, and induced changes in gut microbiota composition. Analysis of EAE affected nerves and muscles showed that VBC treatment significantly decreased their nuclear densities at the disease onset, while nuclear densities of both examined tissues were increased at the end of the disease. Additionally, in popliteal lymph nodes of VBC-treated EAE rats, fewer lymphoid follicles and smaller epithelioid granulomas were observed. Regarding gut microbiota analysis, members of Prevotellaceae family varied most prominently, indicating the potential role of this bacterial family in shaping the course of EAE. Based on our results and previously detected association with neurodegenerative diseases, members of the Prevotellaceae appear as a potential therapeutic target for adjuvant VBC treatment of MS. However, further studies are needed to investigate the complex mechanisms of immune modulation by intestinal microbiota, as well as to determine their interplay with therapeutic agents such as vitamins in the pathogenesis of MS and EAE disease processes.

## Data Availability

Not applicable.

## Supplementary Materials

The following supporting information can be downloaded at: https://www.mdpi.com/article/10.3390/nu14061273/s1, Figure S1: Histological examinations of the popliteal lymph node sections of C and CFA control groups; Figure S2: Difference between the size/extent of left (L) popliteal lymph node in comparison with right (R) after EAE induction; Figure S3: Histological examinations of nerve and muscle nuclear density of C and CFA control groups; Table S1: Beta diversity calculated as weighted Unifrac distance between the samples; Table S2: Relative abundance of genera within Prevoteallaceae family in different samples. Letters a–k indicate pairs of samples that were detected as differentially abundant according to the differentially abundant test based on metastat method as implemented in microeco package.
