# Supplementary material for: Vitamin B Complex and Experimental Autoimmune Encephalomyelitis –Attenuation of the Clinical Signs and Gut Microbiota Dysbiosis
Source: Nutrients. 2022 Mar 17;14(6):1273. doi: 10.3390/nu14061273 (PMC8955508; doi:10.3390/nu14061273)
Supplement: Supplementary file 1 [file nutrients-14-01273-s001.zip › nutrients-1612887-supplementary.pdf]

Supplementary materials

Vitamin B complex and experimental autoimmune encephalomyelitis – Attenuation of the clinical signs and gut microbiota dysbiosis

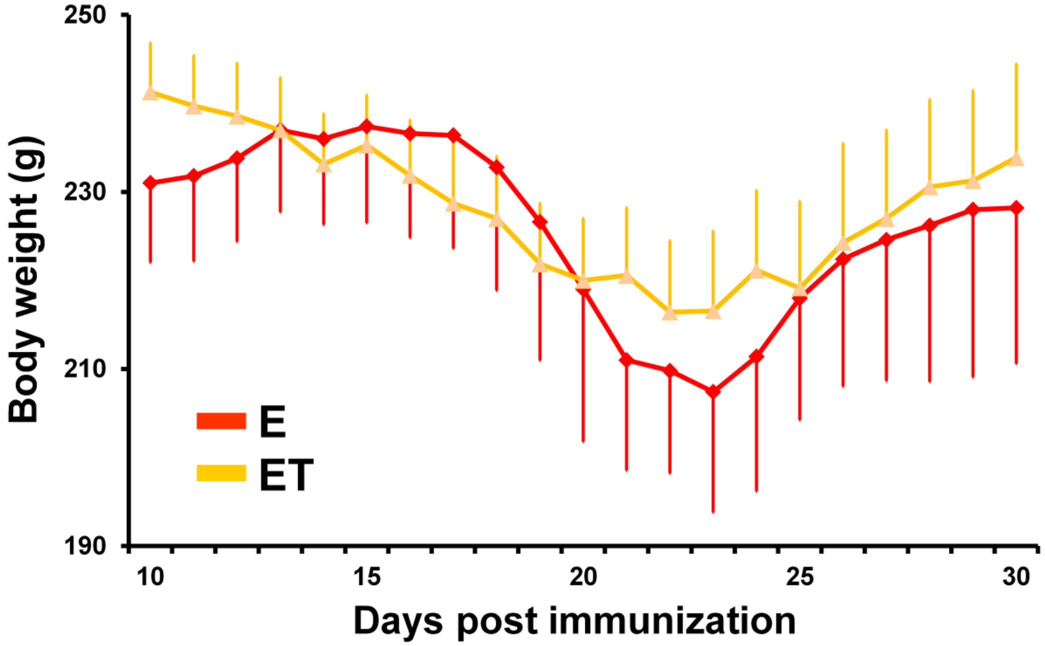

Figure S1. Body weight of untreated (E) and VBC-treated (ET) EAE rats during the disease course. Results are presented as mean ± SE.

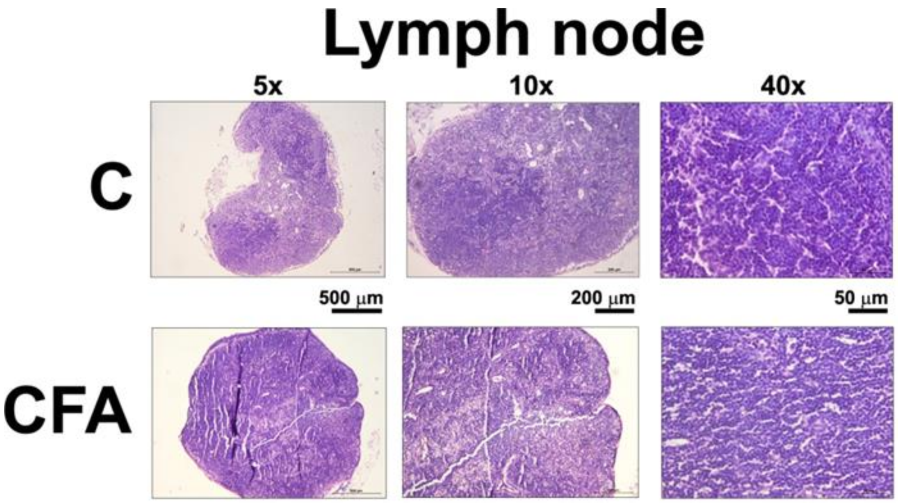

Figure S2. Histological examinations of the popliteal lymph node sections of C and CFA control groups. Micrographs of transverse sections of H&E stained lymph nodes. Magnification 5x (scale bar 500 µm), 10x (scale bar 200 µm), and 40x (scale bar 50 µm).

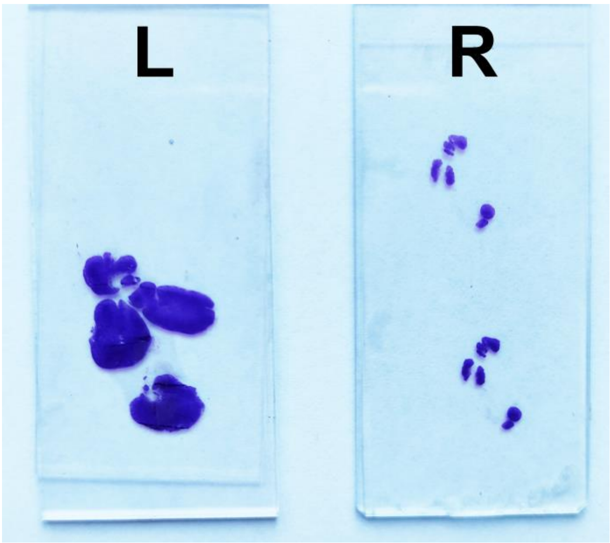

Figure S3. Difference between the size/extent of left (L) popliteal lymph node in comparison with right (R) after EAE induction.

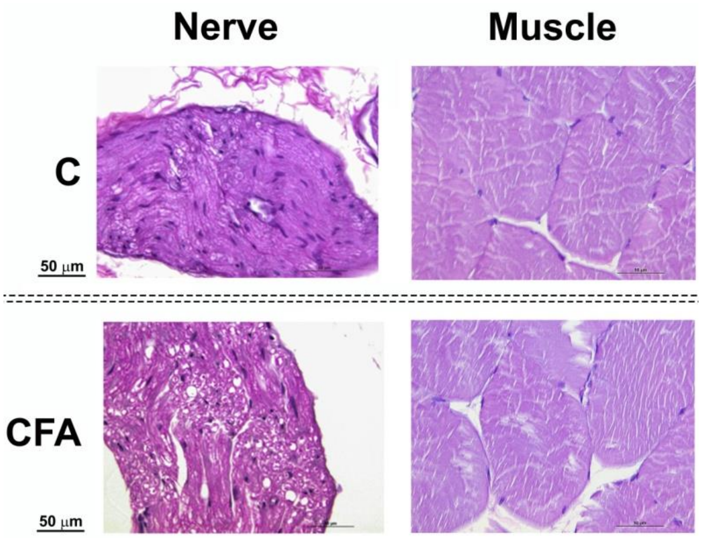

Figure S4. Histological examinations of nerve and muscle nuclear density of C and CFA control groups. Magnification 40x (scale bar 50 μm).

Table S1. Beta diversity calculated as weighted Unifrac distance between the samples.

	C	CFA	Eo	Ep	Ee	ETo	ETp	ETe
**C**	0	0.173535	0.284747	0.284667	0.19407	0.326013	0.246003	0.159983
**CFA**	0.173535	0	0.186428	0.1637	0.164375	0.225354	0.163207	0.10299
**Eo**	0.284747	0.186428	0	0.14472	0.18184	0.202293	0.160026	0.187298
**Ep**	0.284667	0.1637	0.14472	0	0.211488	0.167999	0.150631	0.187482
**Ee**	0.19407	0.164375	0.18184	0.211488	0	0.209439	0.189718	0.115997
**ETo**	0.326013	0.225354	0.202293	0.167999	0.209439	0	0.199031	0.210075
**ETp**	0.246003	0.163207	0.160026	0.150631	0.189718	0.199031	0	0.135509
**ETe**	0.159983	0.10299	0.187298	0.187482	0.115997	0.210075	0.135509	0

Table S2. Relative abundance of genera within Prevoteallaceae family in different samples. Letters a-k indicate pairs of samples that were detected as differentially abundant according to the differentially abundant test based on metastat method as implemented in microeco package.

Genus/Groups	C	Eo	Ep	Ee	$E_{average}$	ETo	ETp	ETe	$ET_{average}$
**Prevotellaceae NK3B31 group**	0.2125[a,b,c,d,e]	0.0081[a]	0.0088[b]	0.1476	0.1476	0.0084[c]	0.0096[d]	0.0843[e]	0.0341
**Prevotella_9**	0.1415 [f,g,h]	0.0855	0.0305[f]	0.055[g]	0.0570	0.0400[h]	0.0688	0.0960	0.0683
**Prevotellaceae UCG-001**	0.0682	0.0226	0.0184	0.0527	0.0312	0.0240	0.0313	0.0500	0.0351
**Prevotellaceae UCG-003**	0.0456[i]	0.0305	0.0261[i]	0.0261	0.0276	0.0261	0.1429[e,h]	0.0514	0.0735
**Alloprevotella**	0.0335[j]	0.0654[j,k]	0.0292	0.0232	0.0393	0.0125	0.0464	0.0449[k]	0.0346
**Prevotella**	0.0199	0.0085	0.0034	0.0075	0.0065	0.0074	0.0116	0.0109	0.0100
**f_Prevotellaceae_g_NA**	0.0108	0.0055	0.0081	0.0078	0.0071	0.0070	0.0098	0.0100	0.0089
**Prevotellaceae Ga6A1 group**	0.0017	0.0025	0.0062	0.0053	0.0047	0.0023	0.0052	0.0015	0.0030
